# Miscellaneous Notices

**Published:** 1854-01-01

**Authors:** 


### Miscellaneous Notices.

*Six Lectures on Materia Medica, and its relations to the Animal Economy.* Delivered before the Royal College of Physicians in 1853. By John Spurgin, M.D., F.R.C.P.S. Churchill. 1853.

Among the able works that have recently issued from the English press on the subject of Materia Medica, there is, to our apprehension, no one to equal in importance the volume before us. Dr. Spurgin has evidently devoted much time and labour to the composition of these lectures, and the result is, that he has produced one of the most philosophical essays on the subject of *Materia Medica* existing in the English language. We regret that our want of space prevents our placing before our readers an analysis of the volume. We would particularly direct the attention of our readers to Dr. Spurgin's views respecting the *modus operandi* of various medicinal agents of known efficacy, and to the chapter appropriated to the investigation of the microscopic character of the blood. The work reflects great credit upon its learned author, and it should find a place in the library of every medical man anxious to keep pace with the progress of medical science.

*Clinical Lectures on Pulmonary Consumption.* By Theophilus Thompson, M.D., F.R.S., Physician to the Hospital for Consumption, &c. &c. London: J. Churchill. 1854.

It is refreshing to take up a volume like the one before us, after perusing the many *ad captandum* works that are yearly published in this country on the subject of consumption. The author of this work has for many years, in fact since its foundation, taken an active part in the establishment of the Brompton Consumptive Hospital. Prior to the erection of this institution, Dr. Thompson had for many years devoted much time to the investigation of pulmonary affections, and the volume now under review may be considered a resumé of his practical knowledge of the treatment of this class of affections. There are many points of psychological interest in Dr. Thompson's work which we had marked for quotation. We refer particularly to his observations on the "Mental Condition of the Dying," to the chapter on "Hysterical Conditions Simulating Consumption," and "On the Influence of Mental Depression as the cause of Phthisis." Whilst directing the attention of the psychological physician especially to these portions of Dr. Thompson's volume, we would, at the same time, observe that the whole work should be carefully studied by all medical men who take an interest in the advancement of our knowledge of the treatment of this dire disease. Dr. Thompson writes like a man who has derived his knowledge of this class of affections at the bed-side of the patient. The work is replete with sound, sensible, practical remarks on the nature and treatment of consumption, and reflects great credit upon the industry, talent, observation, and sagacity of its learned, amiable, and intelligent author. In the next number of our Journal, we hope to place before our readers those parts of Dr. Thompson's volume that relate to psychological phenomena.

*Hufeland's Art of Prolonging Life.* Edited by Erasmus Wilson, F.R.S.

Mr. Erasmus Wilson is entitled to the thanks of the public and profession for this reprint of a work of known ability and reputation.

*On Lithotrity and Lithotomy.* By William Coulson, Esq., Surgeon to St. Mary's Hospital. 1 Vol, 8vo. London: Churchill. 1853.

This volume fully sustains the high surgical character and reputation of its distinguished author. It should be in the library of every practical surgeon.

*Turkey and the Turks.* By the Rev. Archibald Boyd, A.M., Incumbent of Christ Church, Cheltenham. 1853.

This is a lecture delivered to the members of the Church of England Reading Association, and is published at their request, and for the benefit of the Association. We have been much delighted with this charming and intellectual lecture. It is a detailed narrative of a summer tour made this year, 1853, by the author in the East. Mr. Boyd appears to have visited all the points of interest associated with the present unhappy struggle going on in that portion of the globe. His descriptions of the scenery, people, and towns of the East are faithful and graphic; but not more so than we should have expected from the well-known eloquence and literary and classical ability of its accomplished author.

*Highley's Library of Science and Art.* Vol. 1. *The Microscope in its Special Application to Vegetable Anatomy and Physiology.* Highley, Fleet-street. 1853.

Mr. Highley is entitled to the commendation of the scientific world for the publication of this valuable series of useful and philosophical works. The volume before us, on the "Microscope," is written with great care. The author is evidently practically acquainted with his subject, and writes with the skill of a practised hand. In these days of agricultural and botanical progress, this volume should be carefully studied by all interested in such scientific inquiries.

---

